# Glucose-lowering and hypolipidemic activities of polysaccharides from *Cordyceps taii* in streptozotocin-induced diabetic mice

**DOI:** 10.1186/s12906-019-2646-x

**Published:** 2019-08-23

**Authors:** Ru-Ming Liu, Rong Dai, Yi Luo, Jian-Hui Xiao

**Affiliations:** 1 Zunyi Municipal Key Laboratory of Medicinal Biotechnology, Center for Translational Medicine, Affiliated Hospital of Zunyi Medical University, 149 Dalian Road, Zunyi, 563003 People’s Republic of China; 2 The Fourth People’s Hospital of Zunyi, 43 Maanshan Road, Zunyi, 563003 People’s Republic of China

**Keywords:** *Cordyceps taii*, Diabetes mellitus, Glucose-lowering effect, Hypolipidemia, Polysaccharides

## Abstract

**Background:**

Hyperglycemia and dyslipidemia are classic features of patients with diabetes mellitus (DM). *Cordyceps taii*, a folk medicinal fungus native to southern China, possesses various pharmacological activities. This study aimed to assess the glucose-lowering and hypolipidemic effects of polysaccharides from *C. taii* (CTP) in streptozotocin (STZ)-induced diabetic mice.

**Methods:**

Kunming mice were intraperitoneally injected with STZ at a dose of 100 mg/kg body weight. After induction of diabetes, diabetic mice were randomly divided into five groups: diabetic mellitus group (DM), metformin-treated group, low, medium, and high-dose CTP-treated group (CTP-L, CTP-M, and CTP-H). Normal mice served as the control group. After treatment for 28 days, body weight, fasting serum insulin (FSI), fasting blood glucose (FBG), homeostasis model assessment-insulin resistance (HOMA-IR), triglyceride (TG), total cholesterol (TC), low-density lipoprotein-cholesterol (LDL-C), high-density lipoprotein-cholesterol (HDL-C), tumor necrosis factor-α (TNF-α), interleukin-6 (IL-6), and C-reactive protein (CRP) levels were measured. Histological analysis of pancreatic tissue and immune organ indices was also performed to evaluate the anti-diabetes effect of CTP. SPSS (version 21.0) software was used for statistical analysis, and statistical differences were considered significant at *p* < 0.05.

**Results:**

Compared with the DM group, the body weight and FSI level of CTP-H group increased by 36.13 and 32.47%, whereas the FBG and HOMA-IR decreased by 56.79 and 42.78%, respectively (*p* < 0.05). Histopathological examination of the pancreas revealed that CTP improved and repaired the impaired islet β-cells in pancreatic tissue. Compared with the DM group, the levels of TC, TG, and LDL-C decreased by 13.84, 31.87, and 36.61%, whereas that of HDL-C increased by 28.60% in CTP-H (*p* < 0.05). Further study showed that the thymus index in CTP-H was elevated by approximately 54.96%, and the secretion of pro-inflammatory cytokines TNF-α, IL-6, and CRP was inhibited by approximately 19.97, 34.46, and 35.41%, respectively (*p* < 0.05).

**Conclusion:**

The anti-diabetes effect of CTP is closely associated with immunoregulation and anti-inflammation, and CTP may be considered as a therapeutic drug or functional food for DM intervention.

## Background

Diabetes mellitus (DM), a chronic and systemic metabolic disease, is characterized by hyperglycemia and abnormalities in lipoprotein and lipid metabolism; DM can result in a series of complications, such as hypertension, hyperlipidemia, and atherosclerosis [1]. The International Diabetes Federation estimated that approximately 425 million people (20–79 years old) worldwide were afflicted with DM in 2017, and the number will rise to 629 million by 2045 [2]. Thus, DM is considered as a major health risk around the world, and efficient preventive interventions against this disease are essential to improve the quality of life. Insulin and multiple oral hypoglycemic agents, such as metformin, α-glucosidase inhibitors, sulfonylureas, and glibenclamide, are first-line anti-diabetes agents currently in use [3]. These prescribed medications, however, have several drawbacks or side effects, including high cost, occurrence of complications, hypoglycemia, nephrotoxicity, and drug resistance [4]. Therefore, exploring highly efficient, non-toxic, and low-cost novel agents for the treatment of DM and its complications is urgently needed.

Natural products have various advantages, such as diverse structures and bioactivities, and low toxicity, and are regarded as a precious resource for developing new drugs [5]. Polysaccharides are a typical representative of natural products; they have a wide range of sources and possess versatile pharmacological activities, such as anticancer, immunomodulation, antioxidant, antibacterial, and anti-aging effects [6]. In recent years, worldwide interest in the anti-diabetes effects of polysaccharides, particularly polysaccharides from traditional Chinese medicinal herbs, has been increasing because they present promising potential as complementary or alternative medicine for DM [7]. For example, polysaccharides extracted from the lurid bolete mushroom (*Suillellus luridus*) exhibited antihyperglycemic, antihyperlipidemic, and protective effects in STZ-induced diabetic mice [8]. White mulberry (*Morus alba*) fruit polysaccharides displayed good antihyperglycemic and antihyperlipidemic effects, and clearly relieved diabetes symptoms in a T2DM rat model [9]. Thus, natural polysaccharides are one of the most promising avenues for the discovery of new anti-diabetes drugs.

*Cordyceps*, a traditional Chinese medicinal macrofungus, is recognized as a valuable source in the search for natural active polysaccharides and has been used for thousands of years as medicine and food. Previous studies have found that polysaccharides from *Cordyceps cicadae*, *Cordyceps sinensis*, or *Cordyceps militaris* exerted potent anti-diabetes effects [10–12]. *Cordyceps taii* is native to southern China and was first discovered, identified, and named by Professor Zongqi Liang in Guizhou Province in 1991. It has been used as a folk medicine to nourish and strengthen the body in South China [13]. Previously, a variety of small molecular secondary metabolites from *C. taii* have been found to display anticancer activities in vitro and in vivo [14–17].

Our previous study suggested that *C. taii* polysaccharides (CTP), which comprise glucose, galactose, and mannose (molar ratios = 1.14:1.00:1.66) with a series α-(1,4) glucosidic bond, exhibit moderate free radical scavenging ability [18]. Further pharmacological experiments indicated that CTP promoted the production of endogenous antioxidant enzymes, inhibited lipid peroxidation, enhanced the immune system function, and markedly ameliorated oxidative damage in a d-galactose-induced aging mouse model [19]. Thus, CTP is a promising source of natural antioxidant and immunomodulatory agents. Oxygen homeostasis and immune system function are associated with DM [20, 21]. On the basis of the above findings, we hypothesize that CTP has remarkable potential in the treatment of DM. To the best of our knowledge, the anti-diabetes activities of CTP currently remain unknown. Therefore, in the present study, we investigate the anti-diabetes activities and the possible mechanism of action of polysaccharides isolated from *C. taii* by using STZ-induced diabetic mice.

## Methods

### Induction of diabetic mice and treatment protocols

Adult male Kunming mice (6 weeks old) were purchased from the Experimental Animal Center of Third Military Medical University, China (SCXKY 2012–0006). All procedures used in animal experiments were in compliance with the Zunyi Medical University Ethics Committee. The mice were housed in cages at 25 °C ± 2 °C in a 12-h dark–light cycle, with free access to standard pelleted feed and drinking water throughout the experimental period. After 7 days of adapted feeding, the mice were fasted for 12 h but given water ad libitum. Then, the mice were intraperitoneally (ip) injected with STZ (Sigma, USA) dissolved in an ice-cold 0.1 mol/L citrate buffer at pH 4.5 at a dose of 100 mg/kg body weight (bw) and after 4 h, gavaged with 0.2 mL of 5% glucose. Caudal vein blood samples (100 μL) were obtained from STZ-treated mice at 72 h. The mice with fasting blood glucose (FBG) values above 11.1 mmol/L were used as type 1 diabetic models.

After the diabetic state was confirmed, the STZ-induced diabetic mice were divided randomly into five groups (12 mice per group). Normal Kunming mice (12 mice) were served as the control group. All of the animals had free access to water and normal diet. The groups are described in detail as follows:

(i) Control group (CON): normal Kunming mice were treated with normal saline solution. (ii) Diabetic mellitus group (DM): diabetic mice were treated with normal saline solution. (iii) Metformin-treated group (MET): diabetic mice were treated with 100 mg/kg bw of metformin. (iv) Low-dose CTP-treated group (CTP-L): diabetic mice were treated with 100 mg/kg bw of CTP. (v) Medium-dose CTP-treated group (CTP-M): diabetic mice were treated with 200 mg/kg bw of CTP. (vi) High-dose CTP-treated group (CTP-H): diabetic mice were treated with 400 mg/kg bw of CTP.

**Fig. 1:**
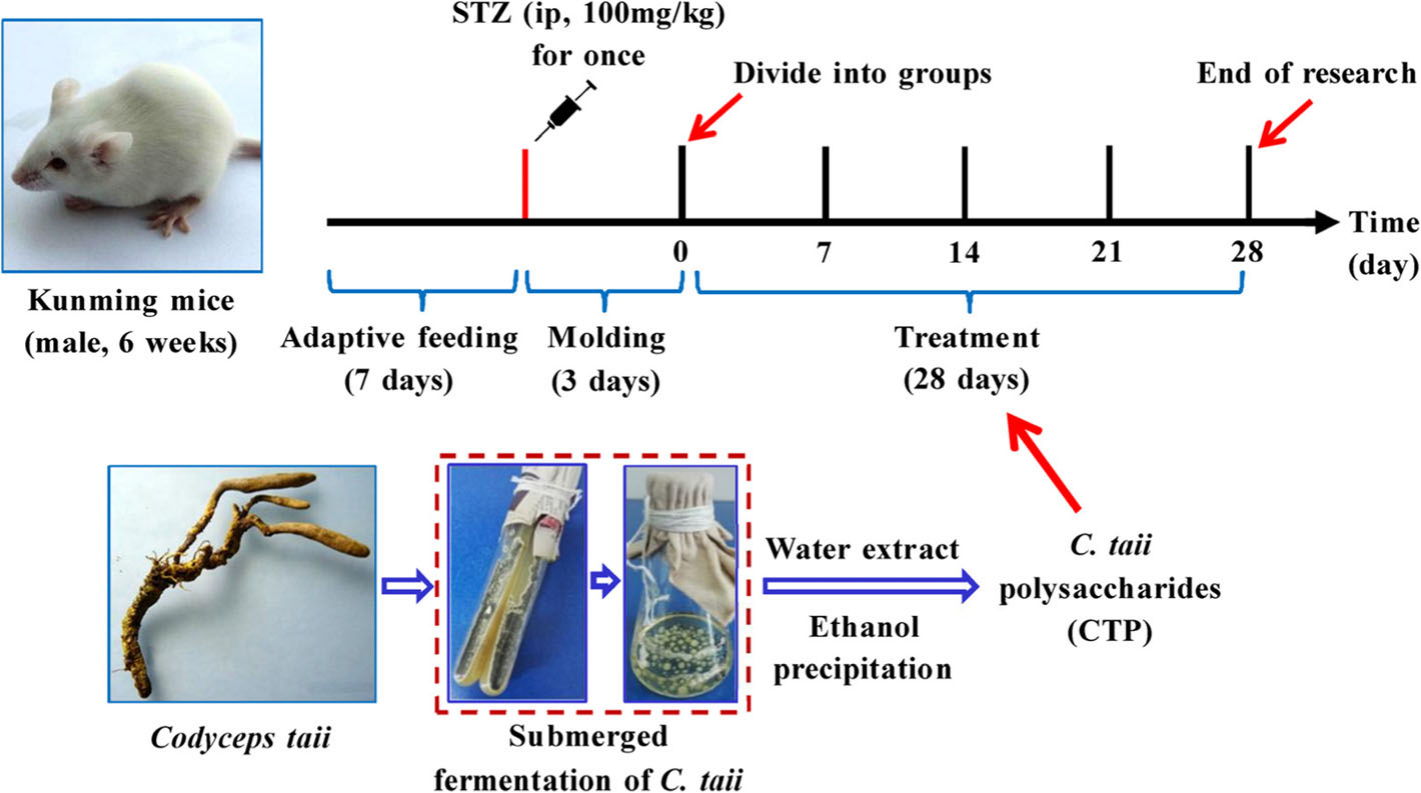
Schematic of the treatment schedule. Adult male Kunming mice (6 weeks old) underwent adapted feeding for 7 days. Except in the CON group, all of the mice were ip injected with 100 mg/kg bw STZ (dissolved in an ice-cold 0.1 mol/L sodium citrate buffer) once. DM mice (fasting blood glucose concentrations higher than 11.1 mmol/L) were randomly divided into five groups after 3 days of modeling: diabetes mellitus group (DM), metformin-treated group (MET), and CTP (CTP-L, CTP-M and CTP-H) group. All of the mice were dosed by gavage once daily for 28 consecutive days

CTP and metformin (Jingfeng, China) were dissolved in 1.0 mL of normal saline. The CON and DM groups received 1.0 mL normal saline respectively. All of the mice were dosed by gavage once daily for 28 days. The metformin and CTP doses were selected on the basis of literature and our previous work [19, 22, 23]. A schematic of the treatment schedule in this research is shown in Fig. 1.

### Sample collection and biochemical analysis

Body weights and FBG levels of mice were measured at one week intervals after CTP was administered. After a 12 h overnight fast, the tail vein blood was used for FBG analysis. On the 28 day, blood samples were collected from the eye vein by quickly removing the eyeball. Then, serum was immediately harvested by centrifuging blood at 900×g for 15 min at room temperature. Samples were stored at − 80 °C for the following assay: serum insulin (R&D Systems, Minnesota, USA), triglyceride (TG), total cholesterol (TC), high-density lipoprotein-cholesterol (HDL-C), low-density lipoprotein-cholesterol (LDL-C), tumor necrosis factor-α (TNF-α), interleukin-6 (IL-6), and C-reactive protein (CRP) levels. The spleen and thymus were removed and weighed for the accession of immune organ indices. The pancreas was removed for histopathological examination.

Plasma glucose was measured using a Roche blood sugar test meter and test strips (Roche Inc., Basel, Switzerland). The insulin, TG, TC, HDL-C, LDL-C, TNF-α, IL-6, and CRP levels in serum were measured by corresponding commercial kits following the manufacturer’s instructions. The serum lipid kits and the inflammatory cytokine kits were purchased from Nanjing Jiancheng Bioengineering Institute (Nanjing, China) and Shanghai Jijin Chemistry Technology Co. (Shanghai, China), respectively. Insulin resistance (IR) was measured with the homeostasis model of assessment-insulin resistance (HOMA-IR) [24]. The HOMA-IR was estimated using the following equation: serum glucose (mmol/L) × serum insulin (mIU/L)/22.5.

### Histopathological analysis of the pancreas

Histopathological examination was performed using standard laboratory procedures. The tissues were embedded in paraffin blocks, and the 4 μm-thick slices were produced and placed onto glass slides. After hematoxylin–eosin (HE) staining, the slides were observed, and images were obtained using an optical microscope.

### Cultivation and mycelia preparation of medicinal fungus

The voucher specimens of *C. taii* (strain GYYA 0601) were deposited at the Laboratory of Institute of Medicinal Biotechnology, Affiliated Hospital of Zunyi Medical University, Guizhou Province, China. The mycelia of *C. taii* were cultured and harvested as previously described [17, 25]. Subsequently, the mycelia were lyophilized and ground (60–100 mesh) for later experiments.

### CTP preparation

CTP was prepared as previously described with slight modifications [18, 19]. In brief, *C. taii* mycelia powder (2 kg) was repeatedly defatted with petroleum ether at 60 °C, and the residue was repeatedly extracted with chloroform and acetic ether (in that order). Subsequently, the residue was extracted with hot water (95 °C) six times (1:10, w/v). The water layers were combined and condensed to one-fifth of their total volume by using a rotary evaporator under reduced pressure at 50 °C and precipitated with three volumes of 95% (v/v) ethanol at 4 °C for 24 h. The precipitate was filtered and dried in an oven at 50 °C for 24 h. The dried crude polysaccharides were dissolved with distilled water, depigmented via the H_2_O_2_ method, deproteinized using the Sevag method, and dialyzed against distilled water for 3 days [19]. The non-dialyzable phase was re-concentrated and fractionally precipitated by ethanol to an 85% final concentration. The resulting precipitate was collected by centrifugation, washed three times with acetone, and dried to a constant weight by vacuum freeze-drying. Finally, CTP was obtained and stored at 4 °C before use. The polysaccharide content was determined using the phenol–sulfuric acid reaction with glucose as a standard. The yield and content of CTP were 3.8% (w/w) and 98.3% (w/w), respectively.

### Statistical analysis

All data are reported as the mean ± standard deviation (SD). SPSS software version 21.0 (SPSS Inc.) was used for all analyses. Statistical analysis was performed by using one-way ANOVA, and post hoc comparisons were processed by Dunnett’s *t*-test. Statistical differences were considered significant at *p* < 0.05.

## Results

### Effects of CTP on body weight in diabetic mice

**Fig. 2:**
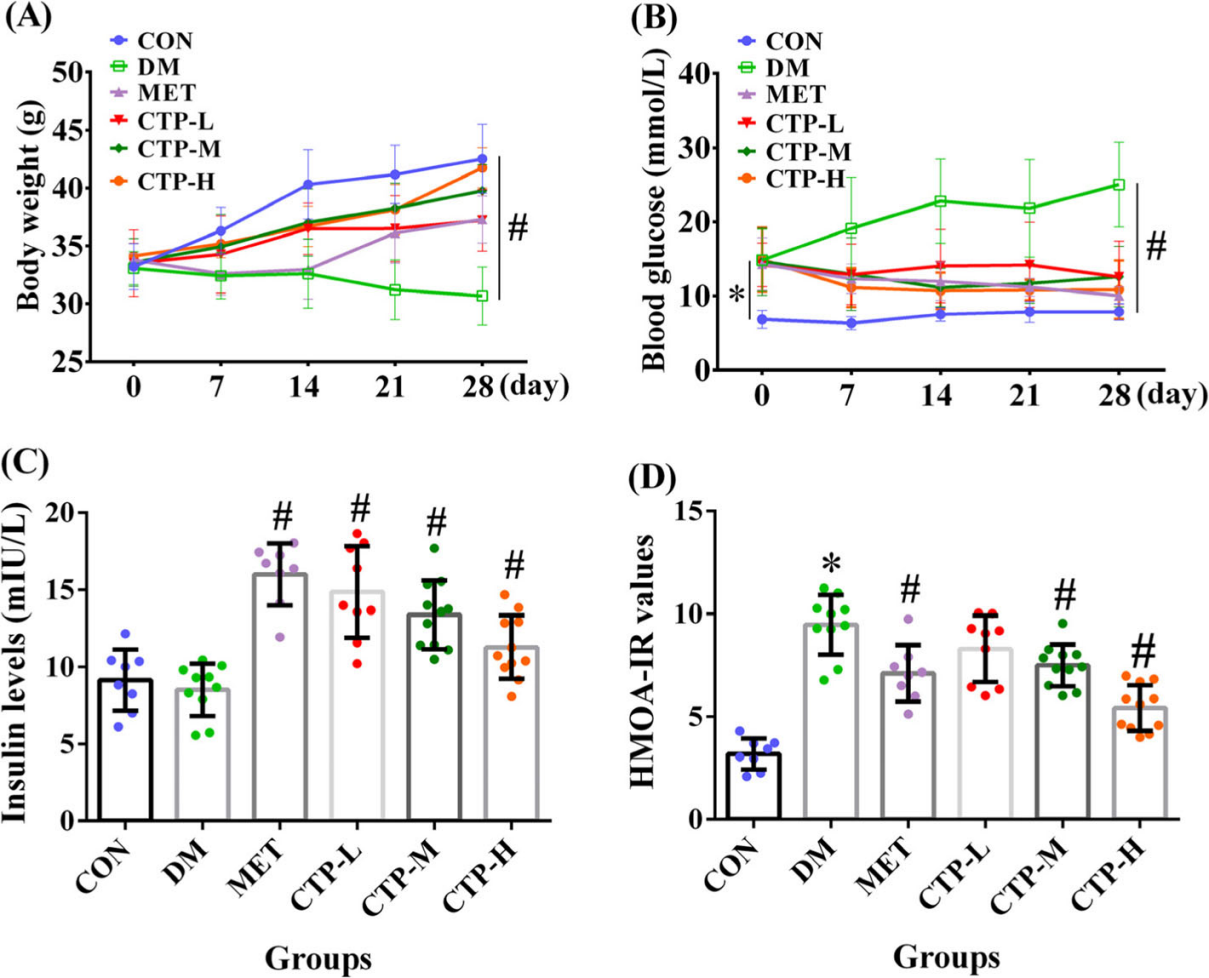
Effects of CTP on body weight, FBG, insulin level, and HOMA-IR value in STZ-induced diabetic mice. **a** Body weight was measured at regular intervals after CTP treatment. **b** FBG level was measured at regular intervals after CTP treatment. **c** Insulin level was measured 28 days after CTP was administered. **d** HOMA-IR value was estimated 28 days after CTP was administered. Data are presented as mean ± SD (*n* = 8–12). * *p <* 0.05 as compared with the normal control group (CON). ^#^ *p <* 0.05 as compared with DM

A decrease in body weight is one of the symptoms of DM. Therefore, the changes in body weight during the experimental period were investigated. As shown in Fig. 2a, the difference in body weights among all of the groups in the initial days was not significant. After 7 days, the body weights of the DM and MET groups were lower than that of the CON group by 10.71% (*p* < 0.05) and 10.13% (*p* < 0.05), respectively. The body weights of the CTP-L, CTP-M, and CTP-H groups were lower than that of the CON group by 5.62, 3.83, and 3.11%, respectively, although all of the results were not statistically different (*p* > 0.05). After 21 and 28 days, the body weights of the MET, CTP-L, CTP-M, and CTP-H groups were significantly higher (*p* < 0.05) than that of the DM group. Among all of the drug-treatment groups, CTP-H showed the greatest weight gain in DM. Thus, CTP treatment resulted in a clear dose-dependent increase in body weights from 100 mg/kg to 400 mg/kg in DM.

### Effects of CTP on FBG and insulin levels and HOMA-IR values in diabetic mice

The FBG, insulin, and IR levels are important evaluation indices for the diagnostic and prognostic evaluation of DM. These indices for STZ-induced diabetic mice were evaluated after CTP treatment. As shown in Fig. 2b, compared with the CON group, all groups tested exhibited an evident increase in glucose over the complete period. However, compared with the DM group, all treatment groups exhibited a significant decrease (*p* < 0.05) in FBG levels at 7, 14, 21, and 28 days posttreatment. Furthermore, compared with the CTP-L and CTP-M groups, the CTP-H group showed a stronger glucose-lowering effect, which was similar to that of the MET group. Also, the effects of CTP on serum insulin levels and HOMA-IR values in diabetic mice are shown in Fig. 2c and d. The results revealed that the oral administration of different concentrations of CTP (CTP-L, CTP-M, and CTP-H) for 28 days to diabetic mice significantly increased the levels of serum insulin (*p* < 0.05) and significantly reduced the level of IR (*p* < 0.05) in a dose-dependent manner compared with the DM group. Compared with the MET group, CTP-H strongly reversed the HOMA-IR value. Thus, CTP reduced the FBG level, increased the insulin level, and decreased the IR of diabetic mice.

### Effects of CTP on serum lipid levels in diabetic mice

**Fig. 3:**
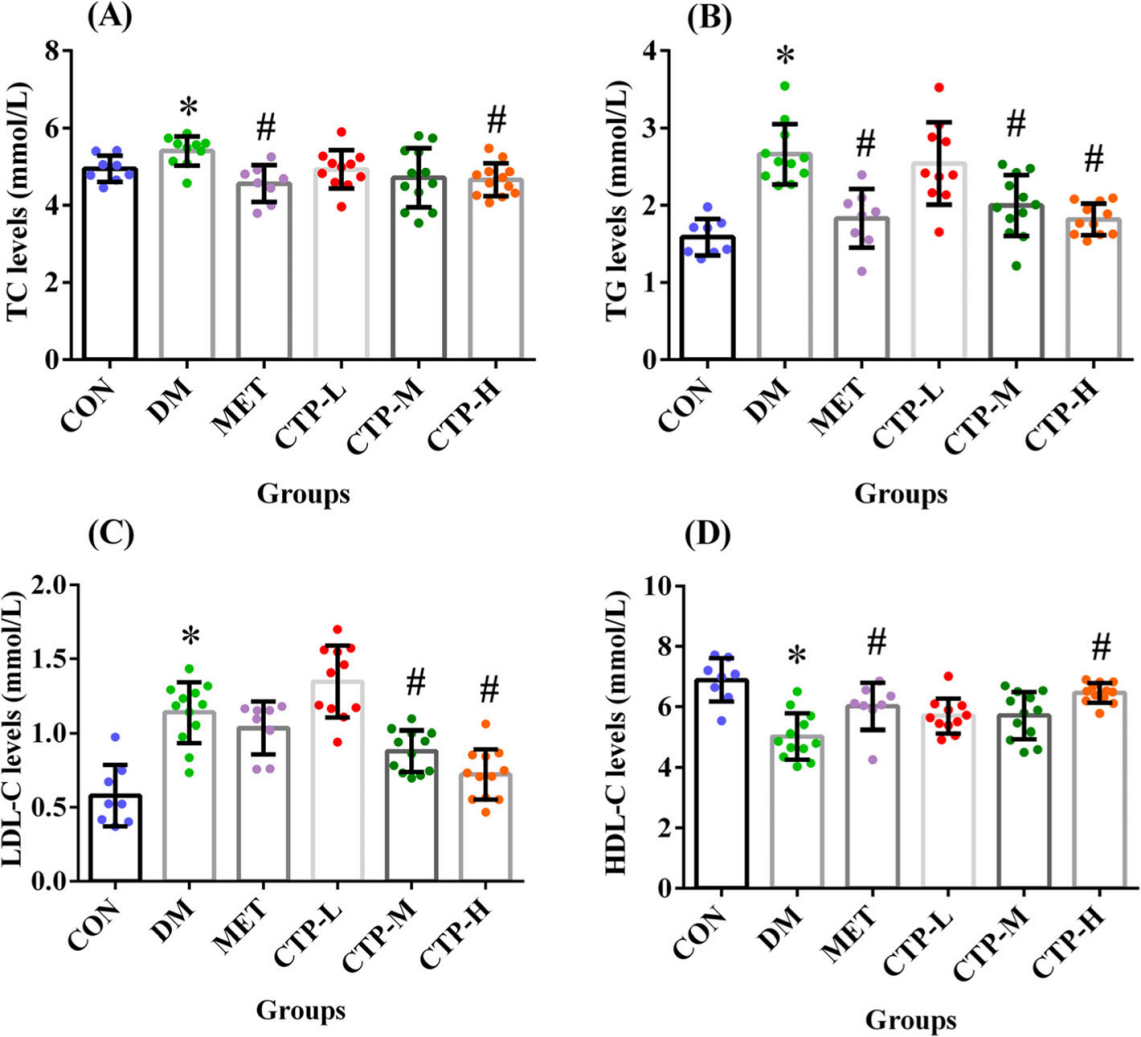
Effects of CTP on serum lipid levels in diabetic mice. The following values were measured 28 days after CTP was administered: (**a**) Serum TC level. **b** Serum TG level. **c** Serum LDL-C level. **d** Serum HDL-C level. Data are presented as mean ± SD (*n* = 8–12). * *p <* 0.05 as compared with CON. ^#^ *p <* 0.05 as compared with DM

DM can also result in lipid metabolism dysfunction. Thus, we further assessed the serum levels of TC, TG, LDL-C, and HDL-C (Fig. 3) in STZ-induced diabetic mice after treatment with CTP for 28 days. As shown in Fig. 3, the TC, TG, and LDL-C levels in the DM group significantly increased (*p* < 0.05), whereas the HDL-C levels decreased (*p* < 0.05) compared with the CON group. Thus, a disorder in blood lipid metabolism occurred in STZ-induced diabetic mice. Similar to the positive control MET, the TC levels of all of the CTP treatment groups decreased in the STZ-induced diabetic mice, although the CTP-L and CTP-M groups did not show statistical differences (*p* > 0.05). The CTP-H group showed significant difference (*p* < 0.05) compared with the DM group (Fig. 3a). Likewise, medium and high doses of CTP decreased the TG levels (Fig. 3b) and significantly decreased the LDL-C levels (*p* < 0.05) compared with the DM group, whereas the positive control MET only exhibited a slight effect (Fig. 3c). Moreover, CTP at different doses enhanced the HDL-C levels, with the most marked effect by CTP-H compared with MET (Fig. 3d). Therefore, CTP could affect lipid metabolic parameters and effectively ameliorate lipid dysregulation in diabetic mice.

### Histopathological observation of the pancreas

**Fig. 4:**
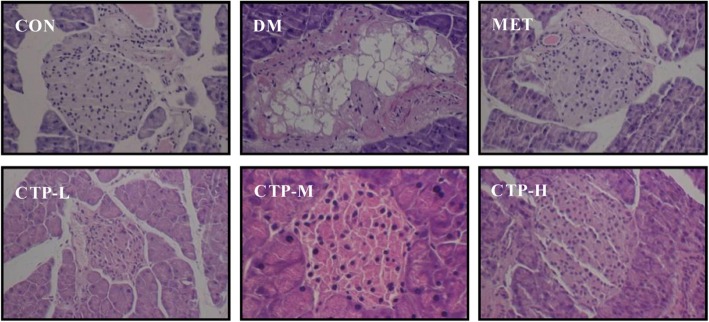
HE staining showing the histopathological changes of the pancreas in diabetic mice treated with CTP for 28 days (200× magnification). The representative data from three independent experiments are shown

The cytoarchitectural changes in the pancreatic tissue were characterized by examining the pathological sections via HE staining (Fig. 4). In the pancreatic samples of normal mice (CON), a complete islet structure exhibiting regularly distributed and abundant pancreatic β-cells was observed. By contrast, the islet cytoarchitecture in STZ-induced mice (DM) showed pronounced endogenous destruction, such as cell rupture, cytolysis, and cavities. Furthermore, the number of pancreatic β-cells was reduced. After treatment with CTP, the abnormal changes in islet structure were rescued in the STZ lesions, contributing to architectural amelioration of islet structure and a gradual increase in β-cell counts.

### Effects of CTP on the immune system and inflammatory cytokines in diabetic mice

**Fig. 5:**
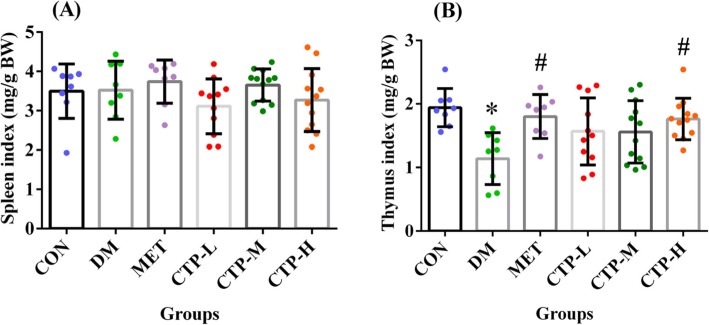
Effects of CTP on immune organ indices in diabetic mice. The following indices were measured 28 days after CTP was administered (**a**) Spleen index. **b** Thymus index. Data are presented as the mean ± SD (*n* = 8–12). * *p <* 0.05 as compared with CON. ^#^ *p <* 0.05 as compared with DM

DM is closely associated with inflammation and the immune system. As the primary immune organs, the spleen and thymus directly affect immune function among organisms [26]. In the current study, the effects of CTP on the spleen and thymus indices of STZ-induced diabetic mice were assessed, as shown in Fig. 5. STZ had no effect on the spleen index in STZ-induced diabetic mice, but the thymus index significantly decreased (*p* < 0.05). Compared with the CON group, the spleen index of all tested groups did not show an obvious statistical difference (Fig. 5a). Similar to the MET group, CTP reversed the thymus index in STZ-induced diabetic mice, and the CTP-H group reached an approximate level similar to that of CON group (Fig. 5b). As such, CTP could restore the thymus index in diabetic mice.

**Fig. 6:**
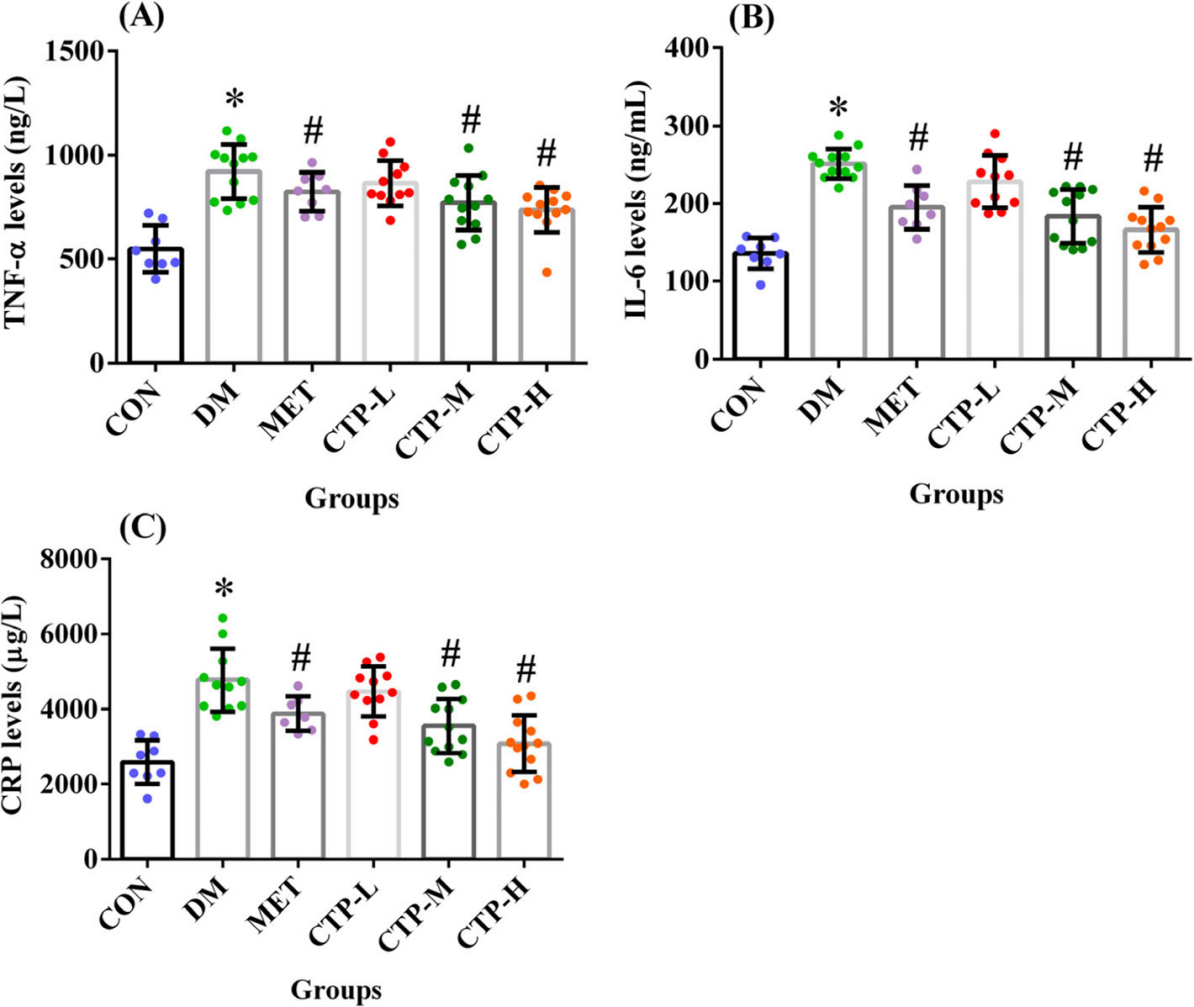
Effects of CTP on the serum inflammatory cytokine levels in diabetic mice. The following values were measured 28 days after CTP was administered (**a**) TNF-α levels. **b** IL-6 levels. **c** CRP levels. Data are presented as the mean ± SD (*n* = 8–12). * *p <* 0.05 as compared with CON. ^#^ *p <* 0.05 as compared with DM

As shown in Fig. 6, we also measured the serum inflammatory cytokines TNF-α, IL-6, and CRP in STZ-induced diabetic mice. The TNF-α, IL-6, and CRP levels in the DM group were significantly increased compared with those in the CON group (*p* < 0.05). Similar to the MET group, medium- and/or high-dose CTP significantly decreased the pro-inflammatory cytokine levels (*p* < 0.05) compared with the DM group. Thus, CTP could inhibit the circulating levels of pro-inflammatory cytokines in diabetic mice.

## Discussion

DM is one of the most common human metabolic diseases. It is characterized by hyperglycemia due to defects in insulin secretion and/or activation, resulting in abnormalities in lipid, carbohydrate, and protein metabolism. The precise cellular and molecular mechanism underlying the etiology and progression of diabetes is still not fully understood.

In recent years, the hypoglycemic and/or hypolipidemic effects of polysaccharides derived from *Cordyceps* species has been frequently investigated. For example, Zhang et al. [10] investigated the anti-hyperglycemic, anti-hyperlipidemic, and antioxidant activities of *C. cicadae* polysaccharide in alloxan-induced diabetic rats. Li et al. [11] found that *C. sinensis* polysaccharide decreased serum blood glucose levels and increased serum insulin levels in STZ-induced diabetic rats and alloxan-induced diabetic mice; *C. militaris* polysaccharide displayed a more prominent hypoglycemic effect than that of *C. sinensis* polysaccharide [12]. Thus, *Cordyceps* polysaccharides are a promising source for developing novel and effective anti-diabetes drugs. However, the structural characteristics and underlying anti-diabetes mechanism for these *Cordyceps* polysaccharides still remain unknown. In the present study, we found that the heteroglycan CTP from *C. taii* possessed good anti-diabetes activity involving glucose-lowering and hypolipidemic activity and reversed IR in STZ-induced DM through a possible immunoregulatory mechanism.

STZ, an antibiotic produced by *Streptomyces achromogenes*, is frequently used to induce DM models for studying the effect of hypoglycemic agents. The mechanism of STZ-induced diabetes includes selective destruction of pancreatic β-cells, which may make cells less active and result in poor sensitivity of insulin for glucose uptake by tissues and hyperglycemia [27]. STZ-induced diabetes is also characterized by severe weight loss, which is possibly because muscle and adipocyte tissues degenerate to compensate for the energy lost [28]. Moreover, at a high single dose, STZ causes massive and rapid β-cells necrosis leading to type 1 DM [29]. At multiple low-doses, STZ leads to partial damage of the β-cells and triggers an inflammatory process [30]. In the present study, based on significantly loss in body weight (Fig. 2a), increase in FBG (Fig. 2b), and destruction of islet cytoarchitecture (Fig. 4), we confirmed that the type 1 diabetic model was successfully constructed with a high single-dose intraperitoneal injection of STZ (100 mg/kg bw) in mice. The results in Fig. 2a showed the significant prevention of loss in final body weight of type 1 DM in the CTP treatment groups. This effect may be the result of glucose-lowering activity of CTP.

FBG and serum insulin levels are important and necessary basal parameters among DM patients [31]. The present study showed that CTP decreased the FBG levels, increased the serum insulin levels and reversed the HOMA-IR values in STZ-induced diabetic mice (Figs. 2). STZ can damage pancreatic β-cells and decrease endogenous insulin secretion, thereby reducing the glucose utilization of tissues. Therefore, the FBG levels in STZ-induced DM decreased because of two reasons. First, CTP restored the damaged pancreatic β-cells (Fig. 4) and increased the pancreatic secretion of insulin. Second, CTP decreased IR (Fig. 2d) or increased the sensitivity to insulin for glucose uptake. However, the underlying mechanism requires further study.

DM as a metabolic disease is associated with abnormal lipid metabolism [32]. Elevated levels of TG, TC, and LDL-C are common in diabetic dyslipidemia, along with lowered HDL-C levels in serum [33]. The abnormal levels of serum lipids among patients with diabetes are primarily attributed to the increased mobilization of free fatty acids from fat deposits inhibiting hormone-sensitive lipase requires insulin [34, 35]. The results in Fig. 3 showed that CTP weakened the levels of TG, TC, and LDL-C and enhanced the levels of HDL-C. Thus, the hypolipidemic effect of CTP may be attributed to the increase in insulin secretion, which increases glucose uptake, inhibits hormone-sensitive lipase and decreases free fatty acids. Additionally, STZ combined with high-fat diet is used to establish type 2 DM, and its characteristics, such as the hyperglycemia and abnormal lipid metabolism, are partly similar to that of STZ-induced type 1 DM except that the weight increases in the former. Recently, Liu et al. [36] found that the aqueous extract of the *Cordyceps militars* fruit body could reduce FBG and decrease blood lipids in diet-STZ-induced type 2 DM rats. Thus, CTP may also exhibit anti-diabetes activity on type 2 DM, and its effect is should be further evaluated through the high-fat diet and STZ-induced mice.

DM is an autoimmune disease [21]. The spleen is the biggest peripheral immune organ, and the thymus is the primary central immune organ [37, 38]. As preliminary indicators, the visceral indices of the spleen and thymus reflect the immune function of the organism [26]. Our previous study suggested that the chloroform extract of *C. taii* could increase the spleen index of Kunming mice bearing sarcoma 180 and the thymus index of C57BL/6 mice bearing melanoma B16F10 [14]. We also proved that CTP increased the thymus and spleen indices in d-galactose-induced aging mice [19]. In the current study, the thymus index of STZ-induced DM increased after treatment with 400 mg/kg of CTP (*p* < 0.05), but the change in the spleen index was not significant (Fig. 5). Accumulating evidence has indicated that DM is accompanied by rapid thymus involution [39]. Chatamra et al. [40] studied thymus atrophy in STZ-induced diabetic rats and reported the reduced numbers of cortical thymocytes and an increase in fibrous tissues. Dagistanli et al. [41] also reported that thymus atrophy is caused by elevated intracellular calcium levels, leading to apoptosis in STZ-induced diabetes.

The thymus is responsible for producing self-restricted and self-tolerant T cells that facilitate and regulate the interaction of lymphoid and non-lymphoid cells [42]. Moreover, DM results from the selective destruction of insulin-producing β-cells in the pancreatic islets and is primarily a T cell-mediated autoimmune disease directed against one or more β-cell autoantigens [43]. Therefore, we speculated that CTP may potentially act as an immunomodulatory agent by restoring thymus weight and enhancing the immune function of pancreatic β-cells in STZ-induced diabetic mice. However, further works are needed to clarify the mechanism, e.g., the numbers of white blood cells and lymphocytes, the precise lymphocyte populations (CD4^+^ T and CD8^+^ T), and the role of regulatory T cells.

Evidence indicates the strong relationship between chronic low-grade inflammation and the pathogenesis of DM [44]. TNF-α and IL-6 are two of the most important pro-inflammatory serum cytokines [45]. High serum levels of TNF-α and IL-6 are associated with IR and DM development, and both cytokines potentially suppress the action of insulin by interfering with the insulin receptor-mediated signal transduction [46, 47]. CRP, an acute-phase protein, is also a strong DM indicator at high levels [48]. The results in Fig. 6 showed that CTP weakened the levels of TNF-α, IL-6, and CRP in the serum of STZ-induced DM. Therefore, our data suggest that the anti-diabetes mechanism of CTP is also associated with repressed chronic inflammation.

The safety of herbal products should be evaluated by using toxicity tests prior to clinical trials. Toxicity should be evaluated to estimate the safe dose and dose range for the clinical trials and identify clinical parameters for potential adverse effects. Therefore, natural polysaccharides should be evaluated for their safety and efficacy for preventing and treating human diseases. *Cordyceps* is relatively safe; as indicated by previous studies on human and animal model toxicity tests, it is safe up 80 g/kg in mice for 7 days and 10 g/kg in rabbits for an extended period of 3 months [49]. Moreover, blood, kidney, and liver functions were normal in models used for the study [50]. Using the 90-day subchronic toxicological assessment, Chen et al. [51, 52] demonstrated that the toxicity of the powder of *C. cicadae* and *C. militaris* submerged mycelial culture was not significant even at the highest dose of 2 and 4 g per kg bw per day in SD rats, respectively. Meena et al. [53] also found that the mycelia powder of *C. sinensis* was safe and non-toxic to rats up to 2 g/kg bw dose. These studies suggest that CTP is safe and non-toxic to mice at an oral dose of 400 mg/kg bw. However, further experiments, that is, standard acute and chronic toxicity and teratogenic tests, are needed to clarify the safety and toxicity of CTP.

In consideration of the limitations of our research, further study should be done to promote the clinical application of CTP. Compared with the STZ-induced type 1 diabetes model, the spontaneously developed type 1 diabetes model, such as non-obese diabetic (NOD) mouse has the pathogenesis similar to the human disease. Therefore, NOD mice can be used to study the precise anti-diabetes mechanism of CTP involved in the steady regulation of immune function and inflammatory cytokine-mediated glycolipid metabolism. Moreover, the clear chemical structure, molecular weight, safety, and toxicity, and pharmacokinetic characteristics of CTP should be clarified.

## Conclusion

We demonstrated for the first time that CTP, a polysaccharide extracted from the mycelium of *C. taii*, displayed potent glucose-lowering and hypolipidemic effects in STZ-induced type 1 diabetic mice. CTP reduced the glucose levels, reversed IR, and ameliorated lipid metabolism. Immunoregulation and anti-inflammation effects may be regarded as possible mechanisms contributing to the anti-diabetes effects of CTP. Additionally, the results suggest that CTP is a potential candidate for developing therapeutic drugs or functional food for treating diabetes and its complications.

## Data Availability

The raw data for this study are available upon reasonable request to the corresponding author.
